# Precisely Tuned Inhibition of HIF Prolyl Hydroxylases Is Key for Cardioprotection After Ischemia

**DOI:** 10.1161/CIRCRESAHA.120.318216

**Published:** 2021-02-25

**Authors:** Aline Jatho, Anke Zieseniss, Katja Brechtel-Curth, Atsushi Yamamoto, Mathew L. Coleman, Ana M. Vergel Leon, Daniel Biggs, Ben Davies, Chris W. Pugh, Peter J. Ratcliffe, Dörthe M. Katschinski

**Affiliations:** 1 Institute of Cardiovascular Physiology, University Medical Center Göttingen, Göttingen, Germany (A.J., A.Z., K.B.-C., A.M.V., D.M.K.).; 2 Nuffield Department of Medicine Research Building, Old Road Campus, Oxford (A.Y., M.L.C., C.W.P., P.J.R.).; 3 Now with Institute of Cancer and Genomic Sciences, University of Birmingham (M.L.C.).; 4 Henry Wellcome Building for Genomic Medicine, Old Road Campus, Oxford, OX3 7BN, United Kingdom (D.B., B.D.).; 5 The Francis Crick Institute, 1 Midland Road, London NW1 1AT, United Kingdom (P.J.R.).; 6 German Center for Cardiovascular Research (DZHK), Partner site Göttingen, Germany (D.M.K.).

**Keywords:** doxycycline, heart failure, incidence, myocardial infarction, prolyl hydroxylase

**Meet the First Author, see p 1121**

Acute myocardial infarction (AMI) is associated with a high incidence of heart failure and mortality. The HIF (hypoxia-inducible factor) system plays a central role in the adaptation to limited oxygen supply and may, in some circumstances, protect against ischemic damage. The protein stability of the HIFα subunits is regulated by 3 oxygen- and iron-dependent PHD (prolyl-4-hydroxylase-domain) enzymes, which are druggable targets.^1^ Roxadustat and daprodustat are first-in-class PHD inhibitors with regulatory approval in China and Japan for renal anemia treatment. They induce endogenous erythropoietin expression by inhibition of all 3 PHD enzymes. Repurposing PHD inhibitors for tissue protection is an attractive strategy, which depends on the possibility of achieving anti-ischemia effects without excessive erythrocytosis. This raises questions regarding PHD isoform-specific effects and timing of treatment. The issue has not been addressed before since the currently available PHD inhibitors lack isoform-specificity. The genetically modified PHD mouse models analyzed so far, either lack the ability to compare PHD isoforms or induce a permanent (sometimes developmental) knockout that does not mimic drug-induced effects in the adult.^2^ To analyze a PHD isoform-specific, temporally determined and reversible response analogous to pharmacological inhibition, we made use of inducible short hairpin RNA (shRNA) interference gene knockdown mice (Figure [A]).^3^ These allow analysis of specific PHD2, PHD1/3, or pan-PHD effects, which due to the CAG promoter however cannot be assigned to a specific cell type or mechanism. Inducing the shRNA expression by doxycycline showed a significant knockdown of the respective target PHD (Figure [B] and [C]). This resulted in a stabilization of HIF-1α and mild to strong activation of HIF-dependent genes in the mice in which PHD2 (Phd2shRNA) and PHD1/2/3 (Phd1/2/3shRNA) were targeted, respectively (although Phd3 induction is suppressed in the latter). In contrast, the expression of HIF target genes was unaffected in the Phd1/3shRNA mice. In the Phd2shRNA mice, the doxycycline dose used produced cardiac effects without increased hematocrit (or apparent leukocytosis/lymphadenopathy),^3^ whereas Phd1/2/3shRNA mice demonstrated a strong erythropoietic effect (Figure [D]). These data confirm that (1) PHD2 is the principal regulator of HIF-1α stability in normoxia and (2) a nonselective inhibition of the PHD enzymes increases the hematocrit.

**Figure.**
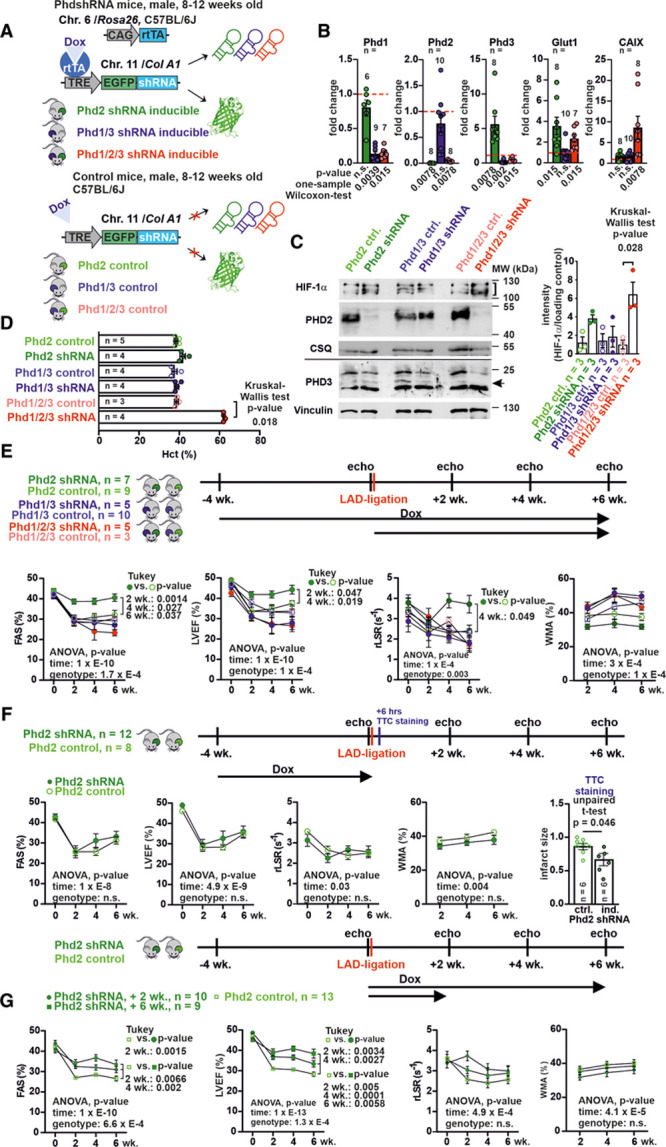
**Postconditional inhibition of PHD2 is essential for cardioprotection after myocardial infarction.** **A**, Schematic of the mouse models. Colors indicate the different alleles encoding the shRNAs. Animal work conformed to institutional guidelines (approval-number 33.9-42501.04.19/3109). **B**, mRNA levels of PHDs (prolyl-4-hydroxylase-domains) and HIF (hypoxia-inducible factor )-target genes in left-ventricles after 4 wk of 0.6 g/l doxycycline (Dox) treatment (in the drinking water) compared with respective control littermates shown as fold change. The analyses include 9, 10, and 6 Phd2, Phd1/3, and Phd1/2/3 control mice, respectively. **C**, PHD2, PHD3, HIF-1α protein levels in the left-ventricles including quantification of the HIF-1α immunoblot signal and **D**, hematocrit (Hct) after 4 wk of 0.6 g/l Dox treatment. **E**, Phd2, Phd1/3 inducible and control mice were treated with 0.6 g/l Dox for 4 wk before and 6 wk after permanent LAD-ligation (ligation of the left anterior descending artery). Phd1/2/3 mice were treated with 0.6 g/l Dox for 6 wk after LAD-ligation. A longer treatment was precluded due to the high Hct. Fractional area shortening (FAS), Left ventricular ejection fraction (LVEF), reverse-longitudinal strain rate (rLSR), and wall motion abnormality (WMA) analyzed by echocardiography (echo) were determined. **F**, Phd2 mice were treated with Dox only in the 4 wk before LAD-ligation. Myocardial infarct size (TTC [triphenyltetrazolium chloride] staining) and cardiac function were analyzed. **G**, Phd2 mice were treated with Dox after LAD-ligation for 2 or 6 wk. After confirming normal distribution, significance was determined by 2-way ANOVA followed by Tukey and unpaired *t* test as indicated. In case of nonparametric analysis 1-sample Wilcoxon or Kruskal-Wallis tests were applied. Chr indicates chromosome; CSQ, Calsequestrin; EGFP, enhanced green fluorescent protein; n.s., not significant; PHD1/2/3, Phd1/2/3shRNA; PHD2, Phd2shRNA; rtTA, reverse tetracycline-controlled transactivator; shRNA, short haipin RNA; and TRE, tetracycline responsive element.

We next employed the shRNA mice to identify which PHD isoform most affects cardiac performance in the context of AMI. Mice were randomly assigned to the experimental groups. Using the doxycycline-inducible shRNA in vivo approach we demonstrate that inhibition of PHD2 for 4 weeks before and 6 weeks after AMI improves fractional area shortening and left ventricular ejection fraction but not diastolic function (reverse-longitudinal strain rate) and wall motion abnormality (Figure [E]). Using the same protocol, Phd1/3shRNA mice did not show any difference in fractional area shortening and left ventricular ejection fraction compared with the control littermates whereas Phd1/2/3shRNA mice performed worse. This was demonstrated by decreased survival (2/5 Phd1/2/3shRNA mice died 4 and 5 weeks after AMI, no death was observed >48 hours postsurgery in the Phd2shRNA, Phd1/3shRNA, or control mice) perhaps contributed to by the elevated hematocrit.

Having demonstrated PHD2 as the primary isoform for cardiac protection, we next approached the question of when PHD2 activity should be inhibited to preserve systolic cardiac performance after AMI. We utilized the on-off-characteristic of the shRNA approach by employing different time-schemes of doxycycline treatment. First, we tested the effect of preconditional inhibition of PHD2 before LAD-ligation (ligation of the left anterior descending artery) (4 weeks). Acute ischemia leads to cardiomyocyte loss, reflected in the infarct size. Previous studies have concentrated on the PHD effects on the acute infarct size and have proposed several protective mechanisms. These include changes in capillary density, NO-mediated vasodilation, metabolic reprogramming, and reduced apoptosis.^4^ In the Phd2shRNA mice, we observed a significantly reduced infarct size but this did not translate into a better cardiac performance (Figure [F]). After the acute phase, the damaged tissue undergoes adaptive remodeling, thereby replacing the mechanically weakened muscle tissue by scar deposition.^5^ Maladaptive remodeling alters ventricular size and composition resulting in impaired cardiac function. Therefore, we tested next the inhibition of PHD2 during this phase via doxycycline treatment for 2 or 6 weeks after AMI (Figure [G]). In this clinically relevant postconditional approach, we showed that a period of 6 weeks and to a lesser extent 2 weeks PHD inhibition after LAD-ligation was sufficient to preserve heart function in terms of improved fractional area shortening and left ventricular ejection fraction.

Hypoxia is involved in many disease pathways and is of special interest in the context of cardioprotection. To exploit the discovery of PHD enzymes in the development of tissue protection therapies, disease-tailored treatment strategies need to be implemented. The strength of the mouse models employed in this study is that timed and PHD isoform-specific effects can be analyzed. The resulting data demonstrate that the treatment schedule and specific inhibition of PHD2 is essential for achieving cardiac protection without increased erythrocytosis. For preserving heart function after AMI, PHD2-specific inhibitors applied in the postconditional phase should be developed and tested.

## Sources of Funding

This work was supported by the German Research Foundation (DFG Ka1269/13-1 to D.M. Katschinski) and the Wellcome Trust (106241/Z/14/Z to PJR). A.M. Vergel Leon is a fellow of the DFG funded IRTG1816.

## Disclosures

P.J. Ratcliffe and C.W. Pugh are scientific cofounders and hold equity in ReOx Ltd, a university spinout company that seeks to develop therapeutic inhibitors of the HIF hydroxylases. The authors will make available all supporting data and methods within the present article. Additional information will be available upon request to the corresponding author. The other authors report no conflicts.
